# Examination of fully automated mammographic density measures using LIBRA and breast cancer risk in a cohort of 21,000 non-Hispanic white women

**DOI:** 10.1186/s13058-023-01685-6

**Published:** 2023-08-06

**Authors:** Laurel A. Habel, Stacey E. Alexeeff, Ninah Achacoso, Vignesh A. Arasu, Aimilia Gastounioti, Lawrence Gerstley, Robert J. Klein, Rhea Y. Liang, Jafi A. Lipson, Walter Mankowski, Laurie R. Margolies, Joseph H. Rothstein, Daniel L. Rubin, Li Shen, Adriana Sistig, Xiaoyu Song, Marvella A. Villaseñor, Mark Westley, Alice S. Whittemore, Martin J. Yaffe, Pei Wang, Despina Kontos, Weiva Sieh

**Affiliations:** 1 Division of Research, Kaiser Permanente Northern California, CA Oakland, USA; 2 Department of Radiology, Kaiser Permanente Northern California, Vallejo, CA USA; 3 Mallinckrodt Institute of Radiology, Washington University School of Medicine, Saint Louis, MO USA; 4 Department of Genetics and Genomic Sciences, Icahn School of Medicine at Mount Sinai, New York, NY USA; 5 Tisch Cancer Institute, Icahn School of Medicine at Mount Sinai, New York, NY USA; 6 Department of Radiology, Stanford University School of Medicine, Stanford, CA USA; 7 Department of Radiology, University of Pennsylvania Perelman School of Medicine, Philadelphia, PA USA; 8 Department of Diagnostic, Molecular and Interventional Radiology, Icahn School of Medicine at Mount Sinai, New York, NY USA; 9 Department of Population Health Science and Policy, Icahn School of Medicine at Mount Sinai, New York, NY USA; 10 Department of Epidemiology, University of Texas MD Anderson Cancer Center, Houston, TX USA; 11 Department of Biomedical Data Science, Stanford University School of Medicine, Stanford, CA USA; 12 Department of Medicine, Stanford University School of Medicine, Stanford, CA USA; 13 Department of Artificial Intelligence and Human Health, Icahn School of Medicine at Mount Sinai, NY New York, USA; 14 Department of Neuroscience, Icahn School of Medicine at Mount Sinai, New York, NY USA; 15 Department of Epidemiology and Population Health, Stanford University School of Medicine, Stanford, CA USA; 16 Sunnybrook Research Institute and Department of Medical Biophysics, University of Toronto, Toronto, ON Canada

**Keywords:** Breast cancer, Mammography, Mammographic density, Risk factors, Epidemiology

## Abstract

**Background:**

Breast density is strongly associated with breast cancer risk. Fully automated quantitative density assessment methods have recently been developed that could facilitate large-scale studies, although data on associations with long-term breast cancer risk are limited. We examined LIBRA assessments and breast cancer risk and compared results to prior assessments using Cumulus, an established computer-assisted method requiring manual thresholding.

**Methods:**

We conducted a cohort study among 21,150 non-Hispanic white female participants of the Research Program in Genes, Environment and Health of Kaiser Permanente Northern California who were 40–74 years at enrollment, followed for up to 10 years, and had archived processed screening mammograms acquired on Hologic or General Electric full-field digital mammography (FFDM) machines and prior Cumulus density assessments available for analysis. Dense area (DA), non-dense area (NDA), and percent density (PD) were assessed using LIBRA software. Cox regression was used to estimate hazard ratios (HRs) for breast cancer associated with DA, NDA and PD modeled continuously in standard deviation (SD) increments, adjusting for age, mammogram year, body mass index, parity, first-degree family history of breast cancer, and menopausal hormone use. We also examined differences by machine type and breast view.

**Results:**

The adjusted HRs for breast cancer associated with each SD increment of DA, NDA and PD were 1.36 (95% confidence interval, 1.18–1.57), 0.85 (0.77–0.93) and 1.44 (1.26–1.66) for LIBRA and 1.44 (1.33–1.55), 0.81 (0.74–0.89) and 1.54 (1.34–1.77) for Cumulus, respectively. LIBRA results were generally similar by machine type and breast view, although associations were strongest for Hologic machines and mediolateral oblique views. Results were also similar during the first 2 years, 2–5 years and 5–10 years after the baseline mammogram.

**Conclusion:**

Associations with breast cancer risk were generally similar for LIBRA and Cumulus density measures and were sustained for up to 10 years. These findings support the suitability of fully automated LIBRA assessments on processed FFDM images for large-scale research on breast density and cancer risk.

**Supplementary Information:**

The online version contains supplementary material available at 10.1186/s13058-023-01685-6.

## Introduction

Mammographic density, or the extent of the breast that appears radiopaque on a mammogram, is an established breast cancer risk factor [1, 2]. Clinically, breast tissue composition is assessed visually by radiologists and categorized according to the American College of Radiology BI-RADS atlas as: (a) entirely fatty; (b) scattered areas of fibroglandular density; (c) heterogeneously dense, which may obscure small masses; and (d) extremely dense, which lowers the sensitivity of mammography [3]. While BI-RADS density categories are routinely recorded on screening mammography reports because of the potential for dense tissue to mask the presence of breast cancer, quantitative measures are preferred for research studies because they provide more information to improve statistical power, robustness and reproducibility in risk prediction. Early research studies manually assessed breast density from film-screen mammograms [1, 4–6]. Over the last 2 decades, conventional film mammography has been replaced with full-field digital mammography (FFDM), obviating the need to digitize film mammograms prior to application of computer-assisted methods. Several studies have found that quantitative density assessments from FFDM images also are strongly associated with breast cancer risk [7–11].

There are multiple methods for quantitating breast density from FFDMs [12]. One of the most common and established methods used in research is Cumulus, a semi-automated tool that facilitates visual thresholding by a trained reader to segment the dense and non-dense areas of the breast [13]. The requirement for thresholding by a trained reader can be an impediment for conducting large-scale studies of thousands of women. More recently, several fully automated methods have been developed. Two commercial automated and validated tools, Volpara [14] and Quantra [15], estimate volumetric density but require the raw ‘for processing’ FFDM images which are not routinely archived for clinical care. Several commercial tools also are available for automatically quantitating area-based density on processed ‘for-presentation’ FFDM images, including Densitas and DenSeeMammo [16, 17]. This study focused on the Laboratory for Breast Radiodensity Assessment (LIBRA) area-based density assessment tool because it is fully automated and publicly available, and can be used on both raw and processed FFDM images [18]. Briefly, LIBRA delineates the breast region by using edge-detection algorithms and applies fuzzy c-means clustering to partition the breast region into gray-level intensity clusters, which are then aggregated into the final dense tissue segmentation. LIBRA density measures have been reported to be associated with breast cancer risk in small case–control studies [7, 9, 11, 18]. However, additional studies in large cohorts are needed to evaluate associations with long-term breast cancer risk (e.g., 5- or 10-year risk) to further validate this automated tool for use in large-scale breast density studies.

The aim of this study was to examine the association between density measures by LIBRA and long-term breast cancer risk in a large cohort of over 21,000 women undergoing screening mammography by FFDM and followed for up to 10 years. We also compare associations with breast cancer risk obtained using LIBRA density measures with those using Cumulus in the same breast cancer screening cohort.

## Methods

### Setting

This study is ancillary to a genome-wide association study of mammographic density [19]. The parent study included non-Hispanic white female participants of the Research Program in Genes, Environment and Health (RPGEH), who completed a health survey and provided a saliva sample for genotyping and who had a least one archived screening FFDM between 2003 and 2013. The RPGEH was established by the Division of Research, Kaiser Permanente Northern California (KPNC). Briefly, the RPGEH resource enables research on the genetic and environmental determinants of common, age-related complex health conditions. The resource links together surveys, biospecimens and derived data, with longitudinal data from electronic health records (EHRs) on a cohort of approximately 400,000 consenting adult KPNC members. Genome-wide genotyping was performed on DNA extracted from saliva samples of more than 100,000 RPGEH participants enrolled before 2010 [20].

### Mammograms

The EHR was used to identify potentially eligible screening FFDMs on the cohort. Processed FFDMs in the KPNC imaging archive came from 37 different KPNC mammography facilities, with 1–5 machines per facility. Over 90% of FFDM machines were manufactured by Hologic or General Electric (GE).

The study was restricted to the 24,800 non-Hispanic white women with Cumulus density measures who met eligibility criteria for a prior study [16]. Briefly, for those Cumulus analyses, we identified Hologic or GE mammograms closest to and on or after the RPGEH survey date. Assessments included dense area (DA) in cm^2^, non-dense area (NDA) in cm^2^, and percent density (PD), defined as the dense area divided by the total breast area and expressed as a percentage. Cumulus assessments were done in batches by a single trained reader using the left craniocaudal (CC) view for ~ 90% of women; the right CC view was randomly selected for ~ 10% of women to blind the reader to breast cancer history, because the prior study included the right CC view for women with prior breast cancer in the left breast. We excluded women who had bilateral breast cancer, bilateral breast implants, breasts too large to be completely imaged on a single exposure, unreadable images or unavailable images [16].

**Fig. 1:**
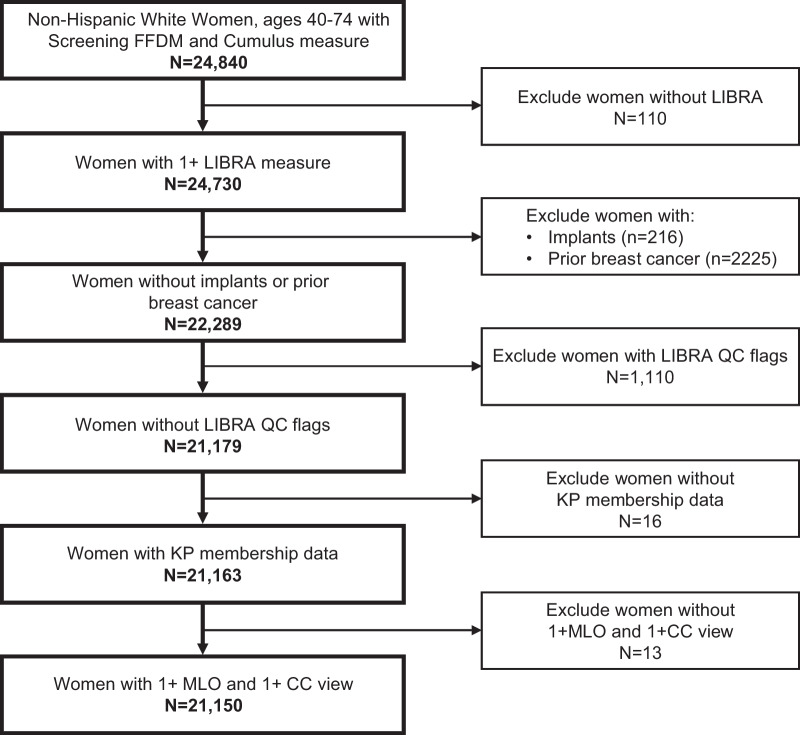
Study cohort eligibility

LIBRA density measures were obtained from the same FFDM exams included in the prior study of Cumulus; however, because assessments are fully automated we analyzed up to 4 breast views instead of just a single right or left CC view. For this current study, we further excluded 110 women for whom a LIBRA measure could not be obtained because of missing data in required DICOM fields, 2225 women with a history of unilateral breast cancer, and 216 women with unilateral implants (bilateral implants and cancer were previously excluded [16]). We also excluded women for the following reasons: their LIBRA values did not pass quality control filters (*n* = 1110) (see Additional file 1: LIBRA quality control steps), they did not have KPNC membership data during the follow-up period (*n* = 16), or they did not have at least one mediolateral oblique (MLO) and one CC view (*n* = 13) (Fig. 1).

### Data sources for cancer diagnoses and covariates

Breast cancer diagnoses were identified from the KPNC cancer registry, which reports to the California Cancer Registry and to the National Cancer Institute’s Surveillance, Epidemiology and End Results (SEER) program of cancer registries. The KPNC registry records information on all new primary cancers (except non-melanoma skin cancer) diagnosed among KPNC members. Data elements and quality assurance measures are similar to SEER. Age at mammogram was determined based on date of birth and date of the mammogram, both from the EHR. We used the body mass index (BMI) from the EHR measured at the patient visit closest to mammogram date. The RPGEH survey provided self-reported information on parity and family history of breast cancer. The KPNC pharmacy database, which records all dispensed outpatient and inpatient prescriptions, was used to determine use of menopausal hormones within the 5 years prior to FFDM.

### Statistical methods

We generated scatter plots and Pearson correlation coefficients to compare density measures from LIBRA vs. Cumulus. Cox regression was used to estimate hazard ratios (HRs) for breast cancer associated with DA, NDA and PD, with time since baseline mammogram as the time scale. Women entered the cohort at the time of their baseline mammogram, and were followed until diagnosis of breast cancer (event) or censored at death, end of KPNC membership or end of study period (12/31/2021), whichever came first. We modeled DA, NDA and PD as continuous variables in units of the standard deviation (SD) in the full cohort. Prior studies have applied different transformations to density measures, including no transformation, log and square-root transformation, [9, 11] so we assessed each of these to increase comparability across studies. Cox regression does not require continuous covariates to be normally distributed, so we did not consider normality when assessing transformations. Since reporting HRs in SD units assumes that the risk increases linearly, we assessed the linearity of the associations for each transformed density measure (Additional file 2: Figure S1). In our final analyses, we used log transformation for LIBRA and Cumulus measures of DA and PD, and untransformed LIBRA and Cumulus measures for NDA. To maximize adjustment for age and BMI (kg/m^2^), these variables were modeled using splines. Parity was categorized as nulliparous, parous, or missing. History of breast cancer in a first-degree family member was categorized as yes or no. The use of menopausal hormones was categorized as none, estrogen alone, or estrogen plus progestin within the 5 years prior to the index mammogram. Analyses of Cumulus density measurements were also adjusted for image batch [19]. Separate multivariable Cox regression models were fit for Hologic and GE mammograms, and the estimates were also combined using random effects meta-analysis. For LIBRA density measurements, separate models were fit for CC and for MLO views, using the average of the right and left views when both were available. We used the average to reduce noise and improve the robustness of our estimates.

## Results

The eligible study population included a total of 21,150 women and 988 incident breast cancer diagnoses within 10 years of follow up (Table 1). There were 17,970 women with a baseline mammogram on a Hologic machine and 3180 women with a baseline mammogram on a GE machine. The distribution of baseline characteristics differed somewhat between the two cohorts, but in both groups most women were between 50 and 70 years of age at baseline mammogram, had no family history of cancer, were parous and had not used postmenopausal hormones in the prior 5 years.

**Table 1:** Baseline characteristics of incident breast cancer cases and the full cohort, by machine type

Characteristic	Hologic				GE			
	Cases		Cohort		Cases		Cohort	
	*N*	%	*N*	%	*N*	%	*N*	%
Total	812	100.0	17,970	100.0	176	100.0	3180	100.0
Age at mammogram								
40–49	43	5.3	1867	10.4	11	6.2	532	16.7
50–59	200	24.6	4837	26.9	48	27.3	965	30.4
60–69	383	47.2	7563	42.1	81	46.0	1265	39.8
70+	186	22.9	3703	20.6	36	20.5	418	13.1
Mammogram year								
2004–2007	60	7.4	695	3.9	42	23.9	571	18.0
2008–2009	284	25.0	5235	29.1	122	69.3	2285	71.9
2010–2011	263	32.4	6605	36.8	12	6.8	300	9.4
2012–2013	205	25.2	5435	30.2	0	0	24	0.8
BMI								
24 or less	283	34.8	7020	39.1	74	42.0	1415	44.5
25–29	281	34.6	5871	32.7	62	35.2	1029	32.4
30–34	137	16.9	3081	17.1	26	14.8	489	15.4
35–39	60	7.4	1246	6.9	8	4.6	168	5.3
40+	51	6.3	752	4.2	6	3.4	79	2.5
First-degree family history of breast cancer								
No	714	87.9	16,498	91.8	155	88.1	2902	91.3
Yes	98	12.1	1472	8.2	21	11.9	278	8.7
Use of postmenopausal hormones^a^								
None	550	67.7	12,401	69.0	95	54.0	1886	59.3
Estrogen alone	94	11.6	2095	11.7	26	14.8	460	14.5
Estrogen + progestin	127	15.6	2287	12.7	49	27.8	563	17.7
Other	41	5.1	1187	6.6	6	3.4	271	8.5
Parity								
Nulliparous	44	5.4	1087	6.0	17	9.7	247	7.8
Parous	645	79.4	13,959	77.7	127	72.2	2308	72.6
Missing	123	15.2	2924	16.3	32	18.2	625	19.6

^a^Within 5 years prior to mammogram date

**Fig. 2:**
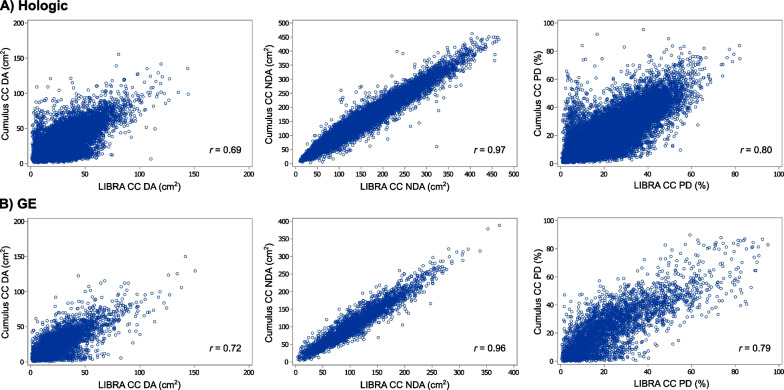
Correlation of dense area, non-dense area, and percent density measurements using LIBRA versus cumulus on full-field digital mammography images acquired from Hologic (**A**) or GE (**B**) machines. LIBRA values were averaged for the right and left cranio-caudal views

Scatter plots of measures from LIBRA (average of left and right views) versus Cumulus (one view, 90% left) on CC views acquired on Hologic FFDM machines show very high correlation for NDA (*r* = 0.97), moderate correlation for DA (*r* = 0.69), and moderate to good correlation for PD (*r* = 0.80) (see Fig. 2). Results were very similar for measures on processed GE FFDM images. While our main results used the average of the left and right views on LIBRA to reduce noise, we also examined correlations of LIBRA and Cumulus measures on the left CC view and found that correlations were very similar to the average of the two CC views on LIBRA. For example, the correlation of LIBRA and Cumulus measures for PD on Hologic FFDM machines was *r* = 0.79 for the left CC view only, and *r* = 0.80 for the average of left and right CC on LIBRA versus left CC on Cumulus.

The fully adjusted HRs for the association between each of the density measures and breast cancer risk are presented in Table 2. Results for LIBRA differed slightly by view, with slightly stronger associations for MLO than for CC views. Results for both LIBRA and Cumulus also differed by machine type, with slightly stronger associations for measures obtained from Hologic than from GE FFDM images. LIBRA results for the MLO view were very similar to Cumulus results, whereas LIBRA results for the CC view were generally weaker than for Cumulus. Results when adjusting only for age and BMI (and batch for Cumulus) (Additional file 3: Table S1) were quite similar to those in Table 2 from fully adjusted models.

**Table 2:** Hazard ratios^a^ per SD of breast density assessments and breast cancer risk, by view and machine type

	LIBRA (MLO^b^ only)	LIBRA (CC^b^ only)	Cumulus
Hologic DA	1.44 (1.33–1.56)	1.28 (1.19–1.38)	1.47 (1.36–1.58)
GE DA	1.24 (1.06–1.46)	1.22 (1.04–1.43)	1.33 (1.11–1.58)
Combined^c^ DA	1.36 (1.18–1.57)	1.27 (1.18–1.36)	1.44 (1.33–1.55)
Hologic NDA	0.85 (0.76–0.94)	0.87 (0.78–0.96)	0.80 (0.72–0.89)
GE NDA	0.85 (0.68–1.07)	0.92 (0.74–1.14)	0.87 (0.70–1.09)
Combined^c^ NDA	0.85 (0.77–0.93)	0.88 (0.80–0.96)	0.81 (0.74–0.89)
Hologic PD	1.52 (1.39–1.67)	1.35 (1.23–1.48)	1.61 (1.47–1.77)
GE PD	1.31 (1.08–1.58)	1.25 (1.04–1.50)	1.39 (1.13–1.70)
Combined^c^ PD	1.44 (1.26–1.66)	1.33 (1.22–1.44)	1.54 (1.34–1.77)

LIBRA and Cumulus DA and PD were log-transformed, and LIBRA and Cumulus NDA were untransformed

^a^Hazard ratios adjusted for age at FFDM (spline), mammogram year (categorical), BMI (spline), parity, first-degree family history, and HRT use within 5 years prior to mammogram date. Cumulus analyses were also adjusted for image batch. HRs are per standard deviation of density based on distribution in full cohort

^b^Average of measures on right and left breasts

^c^Meta-analysis was used to combine Hologic and GE results

HRs for the association of both LIBRA and Cumulus measures with breast cancer risk were generally similar for the periods ≤ 2 years, 2–5 years and 5–10 years after the baseline mammogram, although the inverse association of NDA with risk appeared to be stronger with longer follow-up (Table 3). HRs for the association of both LIBRA and Cumulus measures with breast cancer risk also did not appear to differ markedly when restricting follow-up to ≤ 2 years, ≤ 5 years or ≤ 10 years (Additional file 4: Table S2) similar to results for non-overlapping time intervals (Table 3). For example, the HRs per SD for LIBRA PD (MLO view) were 1.37 (95% CI, 1.08–1.75), 1.43 (1.27–1.62) and 1.45 (1.28–1.65) for ≤ 2 years, ≤ 5 years and ≤ 10 years, respectively.

**Table 3:** Hazard ratios per SD of breast density assessments and breast cancer risk, by time since mammogram

Density measure	≤ 2 years		> 2 to 5 years		> 5 to 10 years	
	HR^a^	95% CI	HR^a^	95% CI	HR^a^	95% CI
LIBRA (MLO^b^ only)						
Dense area (DA)	1.34	1.12–1.61	1.42	1.25–1.61	1.40	1.19–1.65
Non-dense area (NDA)	0.92	0.78–1.08	0.87	0.73–1.03	0.79	0.66–0.93
Percent density (PD)	1.37	1.08–1.75	1.45	1.25–1.69	1.54	1.33–1.79
LIBRA (CC^b^ only)						
Dense area (DA)	1.27	1.12–1.43	1.32	1.16–1.50	1.26	1.11–1.42
Non-dense area (NDA)	0.97	0.82–1.13	0.87	0.74–1.04	0.79	0.67–0.93
Percent density (PD)	1.30	1.12–1.52	1.35	1.16–1.57	1.38	1.19–1.60
Cumulus						
Dense area (DA)	1.44	1.26–1.63	1.43	1.25–1.62	1.48	1.31–1.68
Non-dense area (NDA)	0.86	0.73–1.01	0.88	0.74–1.05	0.72	0.61–0.86
Percent density (PD)	1.55	1.25–1.91	1.46	1.25–1.71	1.67	1.43–1.96

Number of breast cancers for ≤ 2 years, > 2 to 5 years and > 5 to 10 years were 322, 290 and 315, respectively. LIBRA and Cumulus DA and PD were log-transformed, and LIBRA and Cumulus NDA were untransformed

^a^Hazard ratios adjusted for age at FFDM (spline), mammogram year (categorical), BMI (spline), parity, first-degree family history, and HRT use within 5 years prior to mammogram date. Cumulus analyses were also adjusted for image batch. HRs are per standard deviation of density based on distribution in full cohort. Meta-analysis was used to combine Hologic and GE results

^b^Average of measures on right and left breasts

## Discussion

In our study of over 21,000 non-Hispanic white women and nearly 1000 breast cancer cases, we found that LIBRA measures of dense area, non-dense area, and percent density on processed images acquired from both Hologic and GE FFDM machines were associated with breast cancer risk. Moreover, the magnitude of the associations using fully automated LIBRA measures were generally quite similar to that of operator-dependent Cumulus measures. Results for LIBRA measures on the MLO view were the most similar to Cumulus results. We also found that both LIBRA and Cumulus density measures were significantly associated with increased breast cancer risk over a 10-year follow-up period, with similar magnitudes in both the near-term (≤ 2 years) and long-term (5–10 years). These findings provide further evidence that associations of mammographic density with near-term breast cancer risk are not largely explained by masking, and reflect breast tissue characteristics that predispose to future malignant transformation.

Our findings are largely consistent with a few smaller case–control studies that have also reported results on the association between LIBRA density assessments and breast cancer risk with comparison to Cumulus measurements [7, 9, 11]. The first by Busana et al. [11] was a UK study of 414 breast cancer cases and 684 controls, and all FFDMs were acquired on GE machines. They used the CC view only and compared results using processed and raw images. The study found slightly stronger associations for Cumulus than LIBRA measures, and that associations were also slightly stronger for both measures on processed vs. raw images. The adjusted (age, body mass index, menopausal status, parity, age at menarche, ever-use of oral contraceptive and hormonal therapy) OR per SD of DA on processed GE images was 1.39 (95% CI, 1.17–1.64) for LIBRA and 1.53 (95% CI, 1.30, 1.79) for Cumulus, which are fairly similar to our findings for both measures on processed GE images. The second by Nguyen et al. [7] was a Korean study with 398 breast cancer cases and 737 controls, and FFDMs acquired on either Hologic or GE machines. They used the CC view of processed images only and compared results for Hologic and GE machines. In contrast to Busana et al. [11] and to our findings, they found associations were slightly stronger for LIBRA than for Cumulus. For GE, the adjusted (age, BMI, menopausal status) ORs per SD of DA was 1.50 (1.28–1.76) for LIBRA and 1.36 (1.16–1.59) for Cumulus. For Hologic, the adjusted ORs were 1.72 (1.38–2.15) for LIBRA and 1.58 (1.27–1.97) for Cumulus. The third by Gastounioti et al. [9] was a US study of 437 breast cancer cases and 1225 controls, and all FFDMs were acquired on Hologic machines. Density measures for each woman were an average of all four breast views. Similar to Busana et al. [11], they found that associations were slightly stronger for processed than raw images and like Busana et al. [11] and our study, results were slightly stronger for Cumulus than LIBRA measures. On processed Hologic images, the adjusted (age, BMI) ORs per SD of DA were 1.2 (95% CI 1.1–1.4) for LIBRA and 1.3 (95% CI 1.2–1.5) for Cumulus. The present study is the first to provide results for the CC versus MLO views. Our finding that LIBRA measures on the MLO view provide slightly stronger associations with breast cancer risk than the CC view suggests that the MLO views may be preferred, especially if resources limit the number of views available for study. The stronger associations for the MLO view may be related to the initial training of LIBRA, which was done only on MLO views [21]. In addition, our study and the study by Nguyen et al. [7] suggest that associations with LIBRA measures may be slightly stronger on processed images from Hologic vs. GE FFDM machines, although the numbers of GE images in the two studies were relatively small and these findings need to be confirmed by others.

Our study has several strengths and limitations. It is the first large cohort study of automated area-based measures of mammographic density and breast cancer risk and information was available on important risk factors. Given the cohort design and duration of follow-up, we were able to examine associations between density and breast cancer risk in both the near-term (< 2 years) and long-term (5–10 years) periods after the baseline screening mammogram. The associations of LIBRA measures and breast cancer risk were examined by breast view (MLO and CC) and by machine type (Hologic and GE). Cumulus measures were performed by a single radiological technologist and were shown to be strongly associated with breast cancer risk [8]. However, we only had Cumulus measures on the CC view for comparison, although the CC view is the most commonly selected view for Cumulus studies. Given resource constraints, it was infeasible to visually review all images for this study. Instead, we applied a set of quality control criteria that could be automatically implemented to filter out images likely to be incorrectly segmented. Manual review of some of these flagged images, indicated that a small number of correctly segmented images likely were excluded, while some incorrectly segmented images were missed. This quality control process resulted in a slightly higher exclusion percent (5%) than in our Cumulus study (3%) for which all images were visually assessed. Another limitation is that the cohort includes only non-Hispanic white women because it is ancillary to a genome-wide association study [19]. In addition, we did not have raw FFDM images, which are not routinely archived, and could not compare results for raw vs processed images. However, processed FFDM images are more widely available in the clinical setting for use in large-scale studies, and results from studies by Busana et al. [11] and Gastounioti et al. [9] suggest that associations of LIBRA measures with breast cancer risk are stronger on processed than raw images. LIBRA measures on standard two-dimensional FFDM images have been found to be highly correlated with LIBRA measures on synthetic mammograms from digital breast tomosynthesis (DBT) [22], which is increasingly being used in breast cancer screening. However, DBT images were not available for the present study, and future studies will be needed to determine whether density measures on synthetic mammograms are associated with breast cancer risk.

## Conclusions

Our findings, together with the results from other studies, provide substantial support for the use of the fully automated and publicly available density measurement tool, LIBRA, for large-scale studies of mammographic density using processed FFDM images from either Hologic or GE machines. Moreover, LIBRA and Cumulus density measurements were significantly associated with both near- and long-term breast cancer risk and the magnitude of the associations did not attenuate over the 10-year follow-up period. LIBRA allowed us to generate density measures in a fraction of the time and cost needed for a trained reader to measure density using Cumulus. By providing reliable and robust density measures, LIBRA will enable future large longitudinal studies to address multiple questions related to the determinants of mammographic density and its relationship to breast cancer risk, and how these relationships may vary over time.

### Supplementary Information

**Additional file 1**. Quality control steps. LIBRA quality control steps (from D. Kontos and team).

**Additional file 2**. **Figure S1.** Splines of HRs for DA when untransformed, log and square root transformed; splines of HRs for NDA when untransformed, log and square root transformed; Splines of HRs for PD when untransformed, log and square root transformed.

**Additional file 3**. **Table S1**. Hazard ratios* (adjusted for age and BMI only) for breast density assessments and breast cancer risk, by view and machine type.

**Additional file 4**. **Table S2**. Hazard ratios per SD of LIBRA breast density assessments and breast cancer risk, by time since mammogram.

## Data Availability

The datasets analyzed during the current study are available from the corresponding author upon reasonable request.
