# Supplementary material for: Examination of fully automated mammographic density measures using LIBRA and breast cancer risk in a cohort of 21,000 non-Hispanic white women
Source: Breast Cancer Res. 2023 Aug 6;25:92. doi: 10.1186/s13058-023-01685-6 (PMC10405373; doi:10.1186/s13058-023-01685-6)
Supplement: Supplementary file 1 — Additional file 1. Quality control steps. LIBRA quality control steps (from D. Kontos and team). [file 13058_2023_1685_MOESM1_ESM.docx]

**LIBRA quality control steps (from D. Kontos and team)**

1. Selected 4 standard views (i.e. LMLO, LCC, RMLO, RCC) per FFDM study when there was more than one image per view.
   1. If the number of RCC images = LCC images and the number of RMLO images = LMLO images in the study and there was more than 1 image for all 4 views, this is most likely a case of mosaic views due to large breast size. In this case, for each view we selected the image that was acquired first for each mammographic view. Note, most of these women were already excluded in our initial Cumulus study.
   2. Otherwise, this is most likely a case where certain views were repeated due to image artifacts. Therefore, we selected the image that was acquired last for each mammographic view.
2. LIBRA density results
   1. **Extremely low breast total area (TA) values**: Removed images with a breast TA <30 sqcm because this usually indicates incorrect breast segmentations.
   2. **Compare sides within view, looking at breast TA**: Removed studies with breast TAs that differed by >60% when comparing L and R within view. Defined percent difference as |LCC area – RCC area| / minimum (LCC area, RCC area) ×100. (Same equation for MLO.)
   3. **Compare views within side, looking at breast TA**: Removed studies with >100 sqcm absolute difference between LCC and LMLO breast TA or RCC and RMLO breast TA.
   4. **Compare sides within view, looking at DA:** Calculated L vs. R DA differences (i.e., absolute value of difference for DA on the LCC and RCC or LMLO and RMLO).  The CC or MLO difference for each woman on DA was converted to a *Z*-score, or essentially how many standard deviations it is from the mean.  Removed studies with a median *Z*-score of >3 or < -3 because this likely identifies technical issues involving devices, paddles, etc.
   5. **Compare views within side, looking at DA:** Calculated CC vs. MLO DA differences (i.e., absolute value of difference for DA on the LCC and LMLO or RCC and RMLO).  The L or R difference for each woman on DA was converted to a *Z*-score.  Removed studies with a median *Z*-score of >3 or < -3.
   6. **Compare sides within view, looking at PD:** Calculated L vs. R PD differences (i.e., absolute value of difference for PD on the LCC and RCC or LMLO and RMLO).  The CC or MLO difference for each woman on PD was converted to a *Z*-score.  Removed studies with a median *Z*-score of >3 or < -3 because this likely identifies technical issues (devices, paddles, etc).
   7. **Compare views within side, looking at PD:** Calculate CC vs. MLO PD differences (i.e., absolute value of difference for PD on the LCC and LMLO or RCC and RMLO).  The L or R difference for each woman on PD is converted to a *Z*-score.  Removed studies with a median *Z*-score of >3 or < -3.
3. Removed exams when LIBRA PD was >75% and BI-RADS density was 1. Removed exams when LIBRA PD was <25% and BI-RADS density was 4.
