# Supplementary figures and images for: Examination of fully automated mammographic density measures using LIBRA and breast cancer risk in a cohort of 21,000 non-Hispanic white women
Source: Breast Cancer Res. 2023 Aug 6;25:92. doi: 10.1186/s13058-023-01685-6 (PMC10405373; doi:10.1186/s13058-023-01685-6)

**Figure S1.**


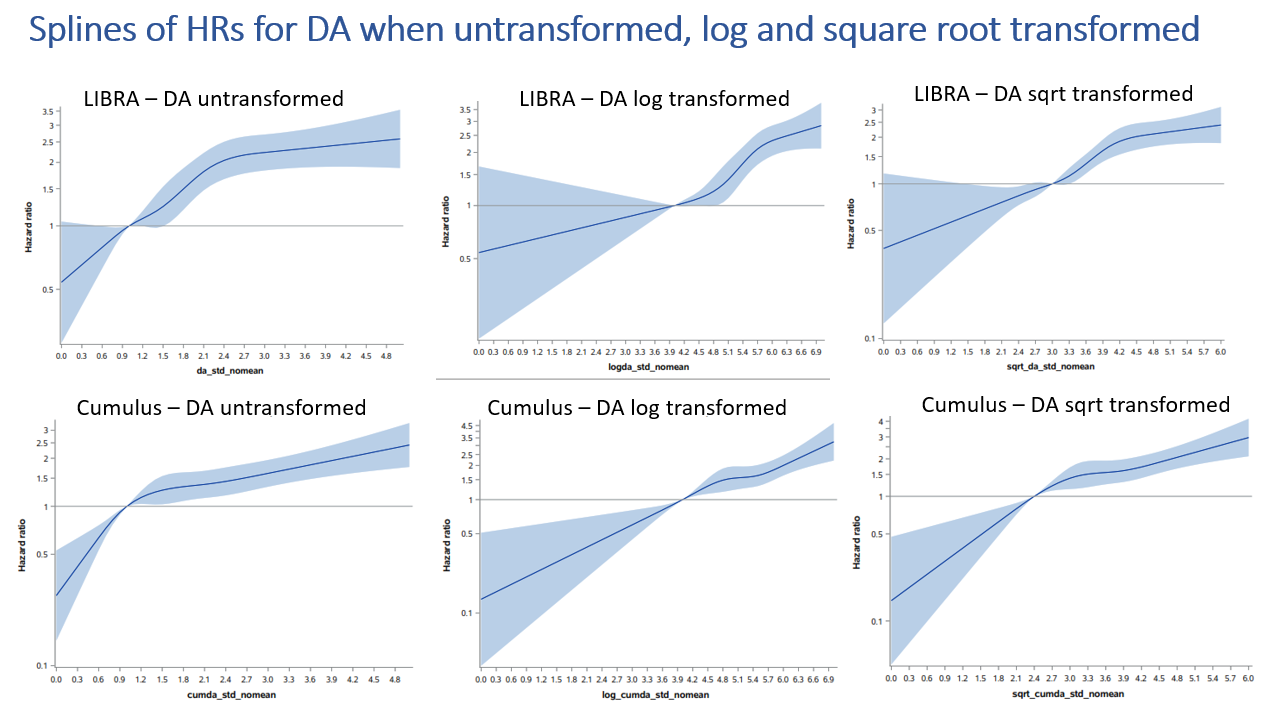


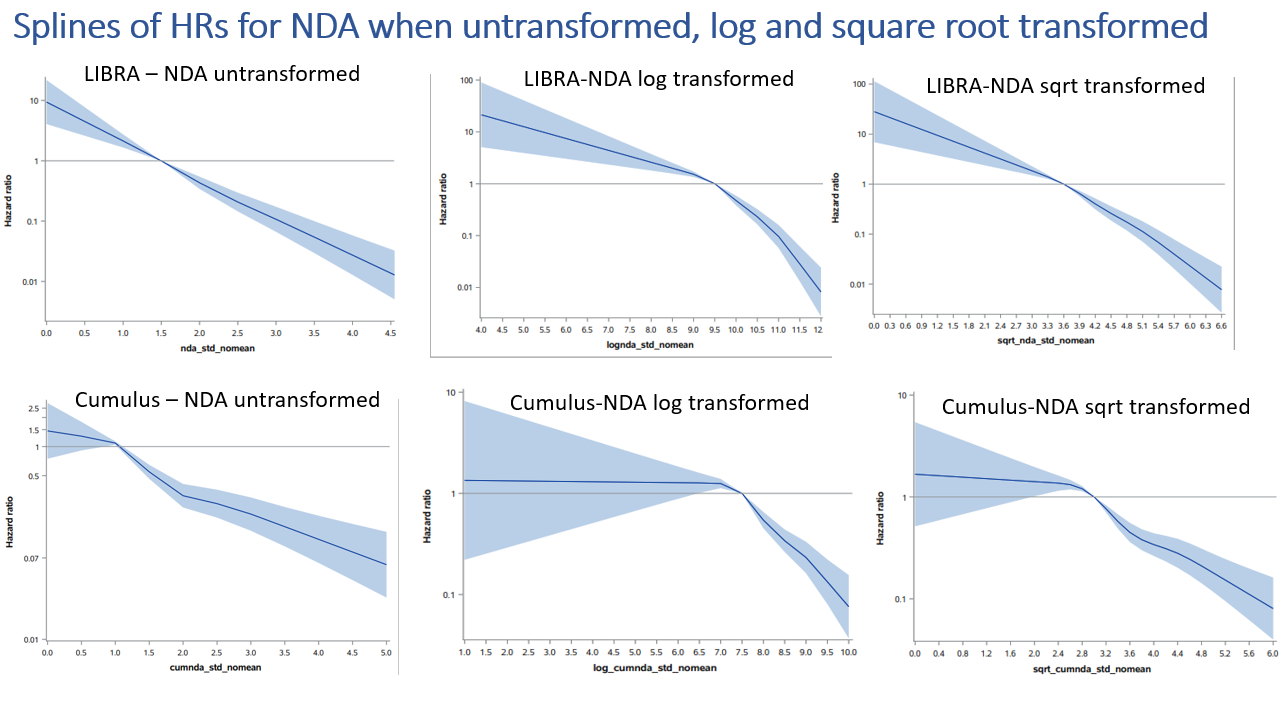


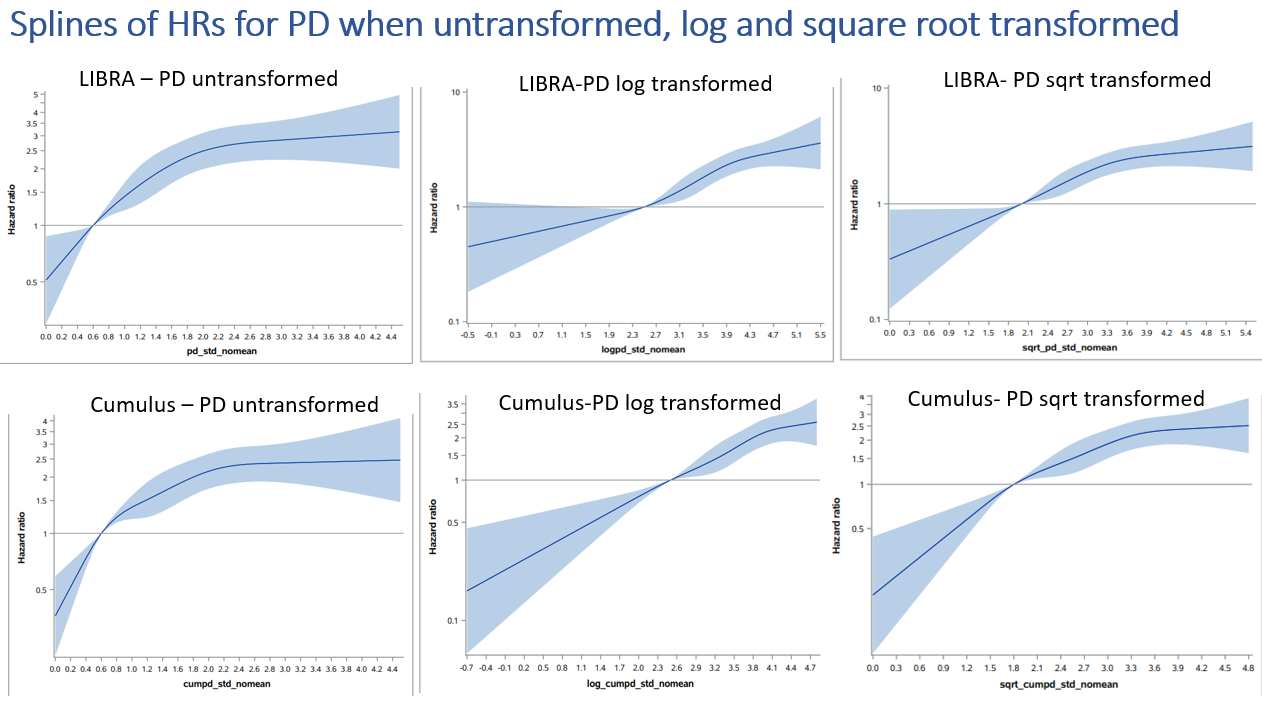

Supplement: Supplementary file 2 — Additional file 2. Figure S1. Splines of HRs for DA when untransformed, log and square root transformed; splines of HRs for NDA when untransformed, log and square root transformed; Splines of HRs for PD when untransformed, log and square root transformed. [file 13058_2023_1685_MOESM2_ESM.docx]
