# Supplementary material for: Examination of fully automated mammographic density measures using LIBRA and breast cancer risk in a cohort of 21,000 non-Hispanic white women
Source: Breast Cancer Res. 2023 Aug 6;25:92. doi: 10.1186/s13058-023-01685-6 (PMC10405373; doi:10.1186/s13058-023-01685-6)
Supplement: Supplementary file 3 — Additional file 3. Table S1. Hazard ratios* (adjusted for age and BMI only) for breast density assessments and breast cancer risk, by view and machine type. [file 13058_2023_1685_MOESM3_ESM.docx]

**Table S1. Minimally adjusted hazard ratios^a^ for breast density assessments and breast cancer risk, by view and machine type.**

|  | **LIBRA (MLO^b^ only)** | **LIBRA (CC^b^ only)** | **Cumulus** |
| --- | --- | --- | --- |
| Hologic DA | 1.46 (1.35-1.58) | 1.30 (1.20-1.40) | 1.48 (1.37-1.59) |
| GE DA | 1.23 (1.05-1.45) | 1.20 (1.03-1.41) | 1.33 (1.12-1.59) |
| Combined^c^ DA | 1.36 (1.15-1.60) | 1.28 (1.19-1.37) | 1.45 (1.33-1.57) |
|  |  |  |  |
| Hologic NDA | 0.84 (0.75-0.93) | 0.86 (0.78-0.95) | 0.80 (0.72-0.89) |
| GE NDA | 0.83 (0.67-1.04) | 0.90 (0.73-1.12) | 0.85 (0.68-1.06) |
| Combined^c^ NDA | 0.84 (0.76-0.92) | 0.87 (0.79-0.95) | 0.81 (0.73-0.89) |
|  |  |  |  |
| Hologic PD | 1.55 (1.41-1.70) | 1.38 (1.25-1.51) | 1.63 (1.48-1.79) |
| GE PD | 1.31 (1.08-1.58) | 1.24 (1.04-1.49) | 1.40 (1.14-1.72) |
| Combined^c^ PD | 1.45 (1.24-1.71) | 1.35 (1.24-1.46) | 1.55 (1.35-1.78) |
| LIBRA and Cumulus DA and PD were log transformed, and LIBRA and Cumulus NDA were untransformed. | | | |

a. Hazard ratios adjusted for age at FFDM (spline) and BMI (spline) only. Cumulus analyses were also adjusted for image batch. HRs are per standard deviation of density based on distribution in full cohort.

b. Average of measures on right and left breasts

c. Meta-analysis was used to combine Hologic and GE results.
