# Supplementary material for: Examination of fully automated mammographic density measures using LIBRA and breast cancer risk in a cohort of 21,000 non-Hispanic white women
Source: Breast Cancer Res. 2023 Aug 6;25:92. doi: 10.1186/s13058-023-01685-6 (PMC10405373; doi:10.1186/s13058-023-01685-6)
Supplement: Supplementary file 4 — Additional file 4. Table S2. Hazard ratios per SD of LIBRA breast density assessments and breast cancer risk, by time since mammogram. [file 13058_2023_1685_MOESM4_ESM.docx]

**Table S2.** **Hazard ratios per SD of breast density assessments and breast cancer risk, by time since mammogram.**

| **Density Measure** | **≤ 2 Years** | | **≤ 5 Years** | | **≤ 10 Years** | |
| --- | --- | --- | --- | --- | --- | --- |
|  | **HR^a^** | **95% CI** | **HR^a^** | **95% CI** | **HR^a^** | **95% CI** |
| **LIBRA (MLO^b^ only)** |  |  |  |  |  |  |
| Dense area (DA) | 1.34 | 1.12-1.61 | 1.38 | 1.22-1.55 | 1.36 | 1.18-1.58 |
| Non-Dense Area (NDA) | 0.92 | 0.78-1.08 | 0.89 | 0.79-1.01 | 0.86 | 0.78-0.94 |
| Percent density (PD) | 1.37 | 1.08-1.75 | 1.43 | 1.27-1.62 | 1.45 | 1.28-1.65 |
| **LIBRA (CC^b^ only)** |  |  |  |  |  |  |
| Dense area (DA) | 1.27 | 1.12-1.43 | 1.29 | 1.19-1.41 | 1.28 | 1.19-1.38 |
| Non-Dense Area (NDA) | 0.97 | 0.82-1.13 | 0.92 | 0.82-1.04 | 0.88 | 0.80-0.96 |
| Percent density (PD) | 1.30 | 1.12-1.52 | 1.33 | 1.20-1.47 | 1.35 | 1.24-1.46 |
| **Cumulus** |  |  |  |  |  |  |
| Dense area (DA) | 1.44 | 1.26-1.63 | 1.41 | 1.24-1.60 | 1.45 | 1.35-1.56 |
| Non-Dense Area (NDA) | 0.86 | 0.73-1.01 | 0.87 | 0.77-0.98 | 0.81 | 0.74-0.90 |
| Percent density (PD) | 1.55 | 1.25-1.91 | 1.48 | 1.24-1.78 | 1.58 | 1.44-1.73 |

Number of breast cancers for ≤2 years, <5 years and <10 years were 322, 612 and 927, respectively. LIBRA and Cumulus DA and PD were log-transformed, and LIBRA and Cumulus NDA were untransformed.

a. Hazard ratios adjusted for age at FFDM (spline), mammogram year (categorical), BMI (spline), parity, first-degree family history, and HRT use within 5 years prior to mammogram date. Cumulus analyses were also adjusted for image batch. HRs are per standard deviation of density based on distribution in full cohort. Meta-analysis was used to combine Hologic and GE results.

b. Average of measures on right and left breasts
